# Supplementary figures and images for: Holoprosencephaly with a Special Form of Anophthalmia Result from Experimental Induction of bmp4, Oversaturating BMP Antagonists in Zebrafish
Source: Int J Mol Sci. 2023 Apr 29;24(9):8052. doi: 10.3390/ijms24098052 (PMC10178349; doi:10.3390/ijms24098052)

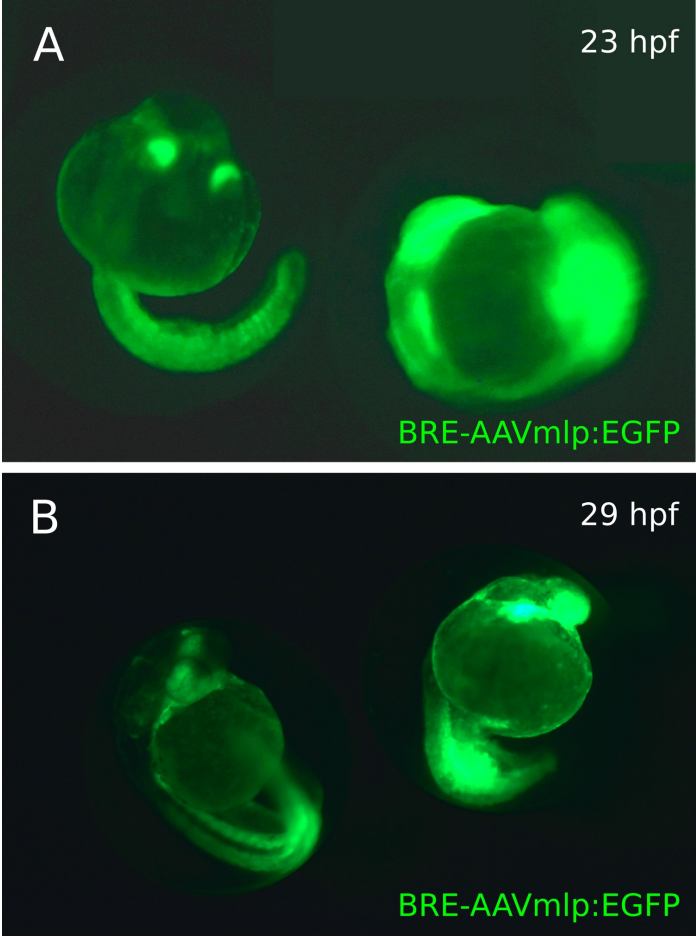

Bulk et al., 2023, Figure S1 Supplemental Information

Supplement: Supplementary file 1 [file ijms-24-08052-s001.zip › ijms-2359744-supplementary.pdf]
